# The complete mitochondrial genome of *Aphis gossypii* Glover, 1877 (Hemiptera: Aphididae) isolated from *Plantago asiatica* in Korea

**DOI:** 10.1080/23802359.2020.1792366

**Published:** 2020-07-20

**Authors:** Yoonhyuk Bae, Jongsun Park, Wonhoon Lee

**Affiliations:** a InfoBoss Inc., Seoul, Republic of Korea; b InfoBoss Research Center, Seoul, Republic of Korea; c Department of Plant Medicine and Institute of Agriculture & Life Science, Gyeongsang National University, Jinju, Republic of Korea

**Keywords:** Mitochondrial genome, *Aphis gossypii*, Aphididae, intraspecific variations, Korea

## Abstract

We have determined mitochondrial genome of *A. gossypii* isolated from *Plantago asiatica* in Korea. The circular mitogenome of *A. gossypii* is 16,045 bp including 13 protein-coding genes, two ribosomal RNA genes, 22 transfer RNAs, and a single control region of 798 bp. Its AT ratio is 83.8%. In comparison this mitogenome to Chinese and Korean *A. gossypii* mitogenomes, 66 single nucleotide polymorphisms (SNPs) and 176 insertions and deletions (INDELs) and 11 SNPs and 173 INDELs are identified, respectively, presenting similar level to those of *Nilaparvata lugens*, *Laodelphax striatellus*, and *Spodoptera frugiperda* and lower than that of *Chilo suppresallis*.

*Aphis gossypii* Glover, 1877 is widely distributed polyphagous and popular pest species to agriculture and horticultural species with influencing mortality of crops and transmitting virus (Ebert and Cartwright 1997). It results that biological control programs for managing Aphid population has been utilized in Europe, Russia, and Korea (Gilkeson and Klein 1981; Vuong et al. 2001). More than 200 species including *Plantago asiatica* (Inaizumi 1970) have been identified as host plants of *A. gossypii* (CABI 2014).

Like previous study that mitogenome of Ophiocordycipitaceae sp. was rescued from the *Ricania speculum* sample (Park et al. 2020), we sequenced the DNA (37°45′74″N, 126°94′84″E; InfoBoss Cyber Herbarium (IN); IBS-00016) prepared from the *P. asiatica* sample with *A. gossypii* extracted using DNeasy Plant Mini Kit (QIAGEN, Hilden, Germany). The sample of *A. gossypii* isolated from *P. asiatica* was identified based on its morphological features. Raw sequences obtained from Illumina HiSeqX (Macrogen Inc., South Korea) were filtered by Trimmomatic 0.33 (Bolger et al. 2014), *de novo* assembled by Velvet 1.2.10 (Zerbino and Birney 2008). Gaps were closed with SOAPGapCloser 1.12 (Zhao et al. 2011), BWA 0.7.17, and SAMtools 1.9 (Li et al. 2009; Li 2013). These works were conducted under the environment of the Genome Information System (GeIS; http://geis.infoboss.co.kr/). Geneious R11 11.1.5 (Biomatters Ltd, Auckland, New Zealand) was used to annotate mitogenome based on Korean *A. gossypii* mitogenome (MN943499; Park Jonghyun et al. 2019).

*A. gossypii* mitogenome (GenBank accession is MT430940) is 16,045 bp long, which is the longer than those of Chinese and Korean *A. gossypii* (Zhang et al. 2016; Park et al. 2019) containing 13 protein-coding genes, two rRNAs, and 22 tRNAs. Its nucleotide composition is AT-biased (A + T is 83.8%). Control region of 784 bp, which is also longer than two *A. gossypii* mitogenomes, is found.

Sixty-six single nucleotide polymorphisms (SNPs) and 176 insertions and deletions (INDELs) and 11 SNPs and 173 INDELs are identified against those of Chinese and Korean *A. gossypii*, respectively. These numbers of sequence variations are similar to those of *Nilaparvata lugens* (Choi et al. 2019; Park, Kwon, et al. 2019; Choi et al. 2020), *Laodelphax striatellus* (Park, Jung, et al. 2019; Seo, Jung, et al. 2019), and *Spodoptera frugiperda* (Seo, Lee, et al. 2019). It is smaller than that of *Chilo suppresallis* (Park, Xi, et al. 2019). One of the possible reasons why our mitogenome is much different from the remaining two mitogenomes, specifically control region, can be a different host plant like the cases of *Acyrthosiphon pisum* (Peccoud et al. 2009) and *Aphis glycine* (Park et al., in preparation).

We inferred the phylogenetic relationship of 23 mitogenomes, including three *A. gossypii* mitogenomes, with one outgroup species, *Bemisia tabaci* (Tay et al. 2016). Multiple sequence alignment was conducted by MAFFT 7.450 (Katoh and Standley 2013). Bootstrapped maximum likelihood, neighbor joining, and Bayesian Inference trees were constructed using MEGA X (Kumar et al. 2018) based on multiple alignment of mitogenomes. The result shows that *Aphis* genus was clearly clustered with the rest genera in Aphidinae, but conspicuously, three phylogenetic trees showed incongruent topology of Aphidinae clade (Figure 1). Taken together, our mitogenome is helpful to understand intraspecific mitogenome variations of *A. gossypii* as well as phylogenetic relationship among Aphidinae species.

**Figure 1.**
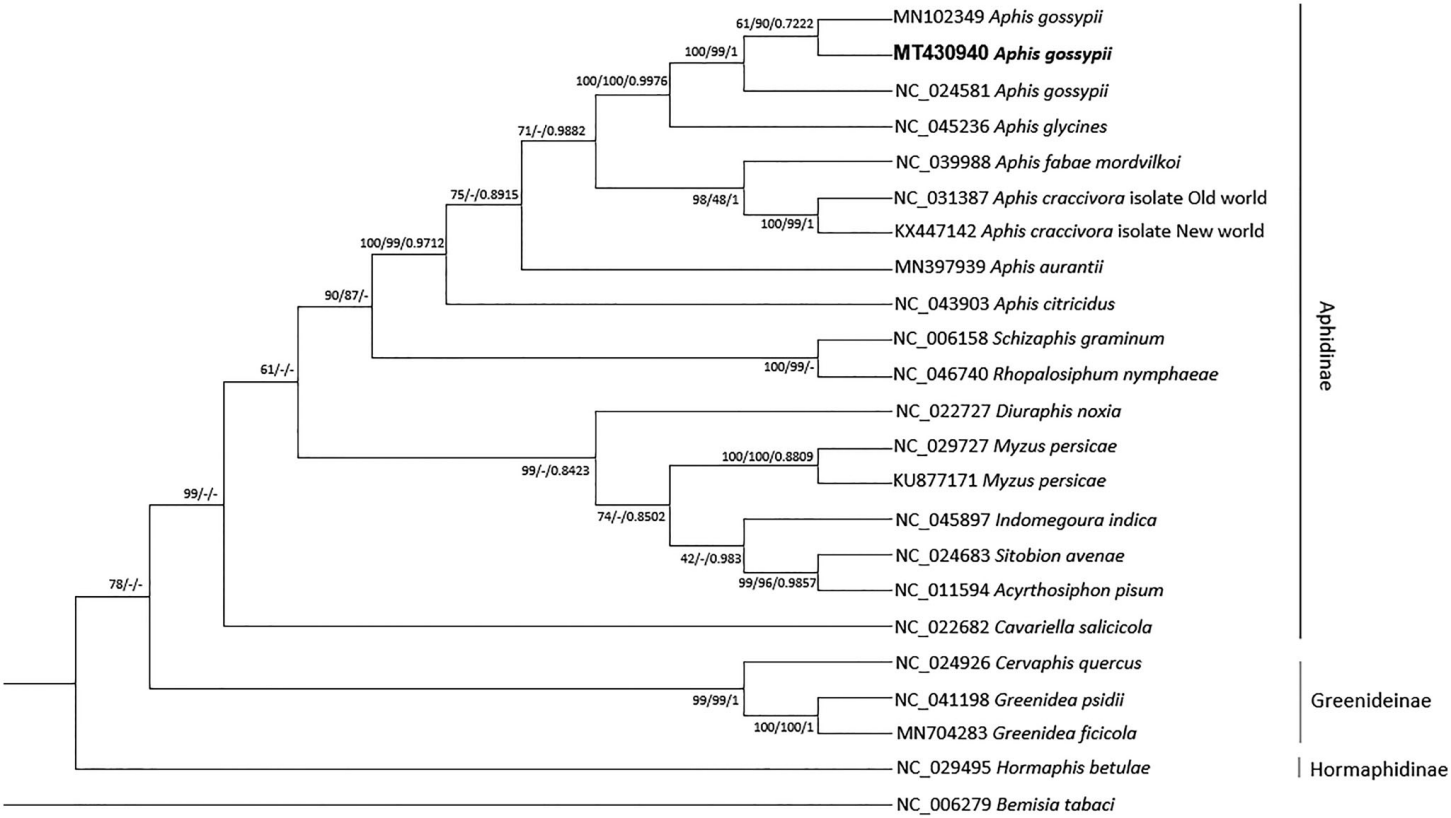
Neighbor joining (10,000 bootstrap repeats), maximum likelihood (1,000 bootstrap repeats), and Bayesian inference (Number of generations is 1,100,000) phylogenetic trees of 23 mitochondrial genomes of Aphididae and one outgroup: three *Aphis gossypii* (MT430940 in this study, NC_024581, and MN102349), *Aphis glycines* (NC_045236), *Aphis fabae mordvilkoi* (NC_039988), *Aphis caccivora* (NC_031387 and KX447142), *Aphis aurantia* (MN397939), *Aphis citricidus* (NC_043903), *Schizaphis graminum* (NC_006158), *Rhopalosiphum nymphaeae* (NC_046740), *Diuraphis noxia* (NC_022727), *Myzus persicae* (NC_029727 and KU877171), *Indomegoura indica* (NC_045897), *Sitobion avenae* (NC_024683), *Acyrthosiphon pisum* (NC_011594), *Cavariella salicicola* (NC_022682), *Cervaphis quercus* (NC_024926), *Greenidea psidii* (NC_041198), *Greenidea ficicola* (MN704283), *Hormaphis betulae* (NC_029495), and *Bemisia tabaci* (NC_006279) as outgroup species. Phylogenetic tree was drawn based on maximum likelihood tree. The numbers above branches indicate bootstrap support values of neighbor joining, maximum likelihood, and Bayesian inference phylogenetic trees, respectively.

## Data Availability

The mitochondrial genomes in this study can be accessed via the NCBI GenBank accession number, MT430940.
